# Strain Background Modifies Phenotypes in the ATP8B1-Deficient Mouse

**DOI:** 10.1371/journal.pone.0008984

**Published:** 2010-02-01

**Authors:** Sohela Shah, Ukina R. Sanford, Julie C. Vargas, Hongmei Xu, Annamiek Groen, Coen C. Paulusma, James P. Grenert, Ludmila Pawlikowska, Saunak Sen, Ronald P. J. Oude Elferink, Laura N. Bull

**Affiliations:** 1 UCSF Liver Center, University of California San Francisco, San Francisco, California, United States of America; 2 Department of Medicine, University of California San Francisco, San Francisco, California, United States of America; 3 Academic Medical Center, Tytgat Institute for Liver and Intestinal Research, Amsterdam, The Netherlands; 4 Department of Pathology, University of California San Francisco, San Francisco, California, United States of America; 5 Department of Anesthesia and Perioperative Care, University of California San Francisco, San Francisco, California, United States of America; 6 Institute for Human Genetics, University of California San Francisco, San Francisco, California, United States of America; 7 Department of Epidemiology and Biostatistics, University of California San Francisco, San Francisco, California, United States of America; University of Florida, United States of America

## Abstract

**Background:**

Mutations in *ATP8B1* (*FIC1*) underlie cases of cholestatic disease, ranging from chronic and progressive (progressive familial intrahepatic cholestasis) to intermittent (benign recurrent intrahepatic cholestasis). The ATP8B1-deficient mouse serves as an animal model of human ATP8B1 deficiency.

**Methodology/Principal Findings:**

We investigated the effect of genetic background on phenotypes of ATP8B1-deficient and wild-type mice, using C57Bl/6 (B6), 129, and (B6-129) F1 strain backgrounds. B6 background resulted in greater abnormalities in ATP8B1-deficient mice than did 129 and/or F1 background. ATP8B1-deficient pups of B6 background gained less weight. In adult ATP8B1-deficient mice at baseline, those of B6 background had lower serum cholesterol levels, higher serum alkaline phosphatase levels, and larger livers. After challenge with cholate-supplemented diet, these mice exhibited higher serum alkaline phosphatase and bilirubin levels, greater weight loss and larger livers. ATP8B1-deficient phenotypes in mice of F1 and 129 backgrounds are usually similar, suggesting that susceptibility to manifestations of ATP8B1 deficiency may be recessive. We also detected differences in hepatobiliary phenotypes between wild-type mice of differing strains.

**Conclusions/Significance:**

Our results indicate that the ATP8B1-deficient mouse in a B6 background may be a better model of human ATP8B1 deficiency and highlight the importance of informed background strain selection for mouse models of liver disease.

## Introduction

ATP8B1, also known as FIC1 (familial intrahepatic cholestasis 1), is an ATP-dependent membrane transport protein in the P-type ATPase family [1]. ATP8B1 belongs to the P4 subfamily of P-type ATPases. Members of this subfamily appear to function in phospholipid transport [2]–[4]; 14 P4 P-type ATPases are encoded in the human genome. ATP8B1 is involved in transport of phosphatidylserine from the outer to the inner leaflet of the plasma membrane [5]–[7]. Mutations in *ATP8B1* result in cholestatic disease with an autosomal recessive mode of inheritance, and ranging in severity from mild and episodic (benign recurrent intrahepatic cholestasis, BRIC1) to chronic and progressive (progressive familial intrahepatic cholestasis; PFIC1) [1], [8], [9]. Patients with severe ATP8B1 deficiency (i.e. PFIC1) typically require liver transplantation prior to adulthood, due to liver failure [10], [11]. While severity and penetrance of ATP8B1 deficiency is correlated with the predicted impact of the *ATP8B1* mutation(s) that a patient carries, additional as-yet-unidentified genetic and/or environmental factors also have an influence [9].

We previously generated mice homozygous for a mutation in *Atp8b1*, the mouse ortholog of *ATP8B1* [12]. These are knock-in mice for the 923G>T point mutation identified in Amish PFIC patients; this mutation results in an amino acid change in a highly conserved residue, G308V [1]. Mice homozygous for this missense mutation are termed *Atp8b1^G308V/G308V^* mice, or ‘ATP8B1 mutant’ mice. These mice exhibited defects in bile acid homeostasis, but did not suffer from progressive cholestatic liver disease. When challenged with a bile salt-supplemented diet, ATP8B1 mutant mice displayed a more severe phenotype, including rapid weight loss, greater liver enlargement, and biochemical evidence of cholestasis, although the phenotype was still less severe than that seen in human patients. Subsequent studies indicated that the canalicular membrane in ATP8B1 mutant mice is susceptible to damage by hydrophobic bile salts, and that hepatobiliary excretion of hydrophobic bile salts is impaired [13], [14].

Published studies have characterized phenotypes in male ATP8B1 mutant mice, and with one exception [14], have focused upon mice in a 129 strain background [12], [13], [15], [16]; preliminary findings suggested that the ATP8B1 mutant phenotype might differ between 129 and C57Bl/6J (B6). Therefore, we compared effects of the *Atp8b1* mutation in 129 and B6 strain backgrounds. Here, we present evaluation of aspects of serum and bile biochemistry, as well as body- and liver-weight related phenotypes, in male and female WT (wild-type) and ATP8B1 mutant mice in B6, 129, and F_1_ (B6×129) strain backgrounds.

## Results

To investigate the simultaneous effects of mutation (WT and ATP8B1 mutant), background strain (B6, 129, and F_1_), sex (male and female), and diet (cholate-supplemented and control), we conducted a factorial experiment. We studied ≥5 mice for each of the (2×3×2×2 = 24) factorial combinations (“full factorial experiment”) [17]. This approach allowed us to study not only the individual effects (“main effects”) of each factor (mutation, diet, genetic background, and sex), but also whether the effect of a factor depended on other factors (“interactions”) (Table 1). Unless otherwise indicated, p-values reported in the text were derived from ANOVA comparing the groups mentioned; when sex differences were not apparent, data from males and females were sometimes combined for these latter tests. For visual clarity, means and standard errors of the mean (SEM) are used to summarize the data in the figures, instead of showing the many p-values from ANOVA.

**Table 1:** Summary of the factorial experiment.

	Main Effects				Interactions					
Phenotypes	Genotype	Strain	Diet	Sex	Genotype x Strain	Genotype x Diet	Genotype x Sex	Strain x Diet	Strain x Sex	Diet x Sex
Pup survival	-	Y	NA	NA						
Mid-nursing weight	Y	-	NA	Y						
Weaning weight	Y	Y	NA	Y						
% weight loss/day	Y	Y	Y	Y	Y	Y		Y		
Baseline serum cholesterol	Y	Y	NA	Y	Y					
Post-diet cholesterol	Y	Y	Y	Y		Y				Y
Baseline serum ALP	Y	Y	NA	Y	Y					
Post-diet ALP	Y	Y	Y	Y		Y	Y			Y
Baseline bilirubin	-	-	NA	-						
Post-diet bilirubin	Y	Y	Y	-		Y				
Baseline serum bile salts	Y	Y	NA	-						
Post-diet serum bile salts	Y	-	+	-		Y				
Post-cholate biliary cholesterol∧	Y	Y	NA	-	Y					
Post-cholate biliary phospholipids∧	Y	Y	NA	-						
Post-cholate biliary bile salts∧	Y	-	NA	-						
% liver weight relative to final body weight #	Y	Y	Y	Y	Y	Y		Y		

The first column lists the phenotypes studied. Columns 2–5 list the main effects of 4 factors- genotype, strain, diet, and sex. Columns 6–11 list the interactions between genotype, strain, diet, and sex. ‘Y’ indicates that a main effect or interaction influences the phenotype. ‘−’ indicates no main effect. ‘+’ indicates that the factor had no main effect, but influences the phenotype when interacting with one or more of the other factors. ‘NA’ indicates that the factor was not included or assessed in the experiment. ∧As more data were available for F1 and B6 mice on cholate, than control, diet, only results of analysis of cholate diet are shown here. #For percent liver weight relative to final body weight, a 3-way interaction was detected between strain, diet, and genotype.

### Pup Survival from the Mid-Nursing Period to Weaning Is Lower in B6 Mice

Offspring of heterozygote couples were less likely to survive from midway through the nursing period (∼day 10) to weaning (∼day 21) if they were of B6, than of 129 or F_1_ background (Table 1; for offspring of all genotypes, p<0.0003 for B6 versus 129 and p<0.0001 for B6 versus F_1_, chi-squared test; this difference, when analyzed separately for mutants and for pooled WT and heterozygotes, remains significant between strains). While 7% of B6 pups died during this period, well under 1% of F_1_ or 129 pups did. Amongst the B6 mice, 14% of mutant, and 5% of pooled WT and heterozygote, mice died during this period (p = 0.065).

### ATP8B1 Mutant B6 Mice Exhibit Slower Weight Gain during the Nursing Period

**Figure 1:**
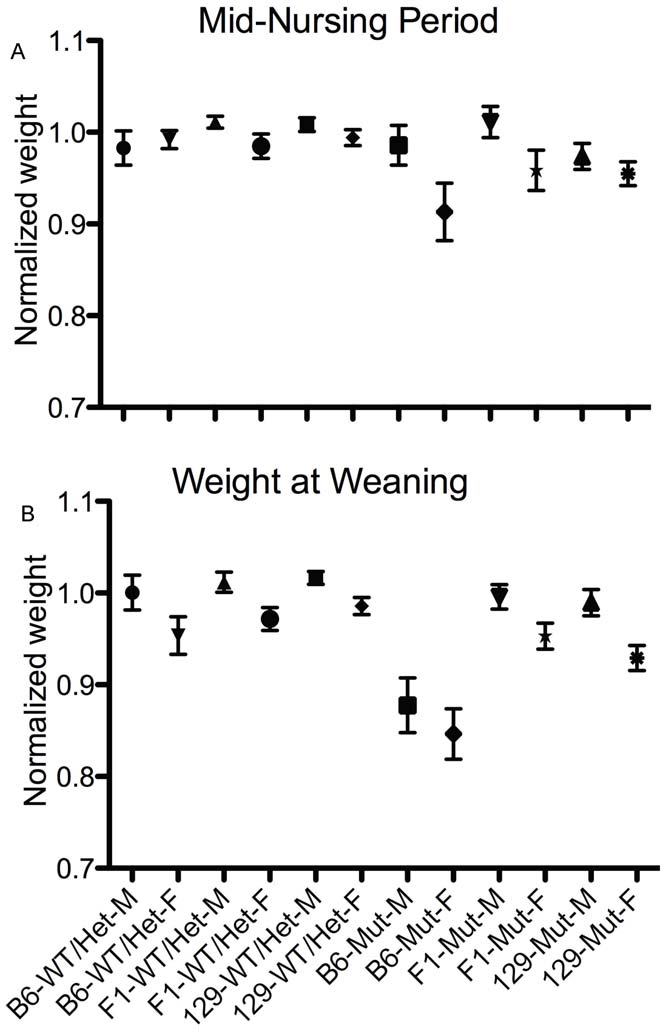
ATP8B1 mutant B6 mice exhibit slower weight gain during the nursing period. Pup weight in WT and ATP8B1 mutant mice of B6, 129, and F_1_ backgrounds at: A) mid-nursing period (∼day 10) and B) weaning. Weights of mutant pups were normalized to those of WT and heterozygote littermates; means and SEM are shown. Range of N's: a) B6 (n = 14−56), 129 (n = 23−57), and F_1_ (n = 70−136) and b) B6 (n = 12−53), 129 (n = 23−58), and F_1_ (n = 50−138).

Previous study had suggested that mutant mice were slightly smaller at weaning than WT and heterozygote littermates [12]. We weighed pups born to heterozygote couples midway through the nursing period and again at weaning. At both timepoints, mutant mice trended smaller than their WT and heterozygote littermates (Table 1, Figure 1 a and b). There was no effect of strain on this difference midway through the nursing period. At weaning, however, B6 mutant mice were 12% smaller than their WT and heterozygous littermates; this mutation-dependent weight difference was greater than that seen in 129 and F_1_ mice (B6 versus 129 and F_1_ males: p<0.01 for both comparisons; B6 versus F_1_ females: p<0.001; B6 versus 129 females: p<0.05). A mutation-associated defect in weight gain during the nursing period thus appears greater in mice of B6 background, as compared to 129 and F_1_ mice.

### The Proportion of Mutant Pups Is Lower than Expected

Overall, among pups born to heterozygote couples, and genotyped at weaning, the mutant allele had a frequency of 46%, which is slightly lower than the expected 50% frequency (p<0.002). This indicates a mild survival benefit conferred by the wild-type allele. The genotype frequencies were in Hardy-Weinberg equilibrium (p = 0.81), and were not observed to differ with strain background (p = 0.85). Consistent with the allelic analysis, 21% of pups born to heterozygote couples, and genotyped at weaning, were mutants. This is modestly lower than the expectation of 25%, indicating a mild decrease in rate of survival to weaning for mutant pups, compared to WT and heterozygote littermates (p<0.01, chi-squared test, N = 861).

#### Diet Studies

Findings were assessed at baseline, and after short-term feeding of a diet supplemented with 0.5% cholate, or a control diet.

### ATP8B1 Mutant B6 Mice Lose Substantial Weight upon Cholate Feeding

**Figure 2:**
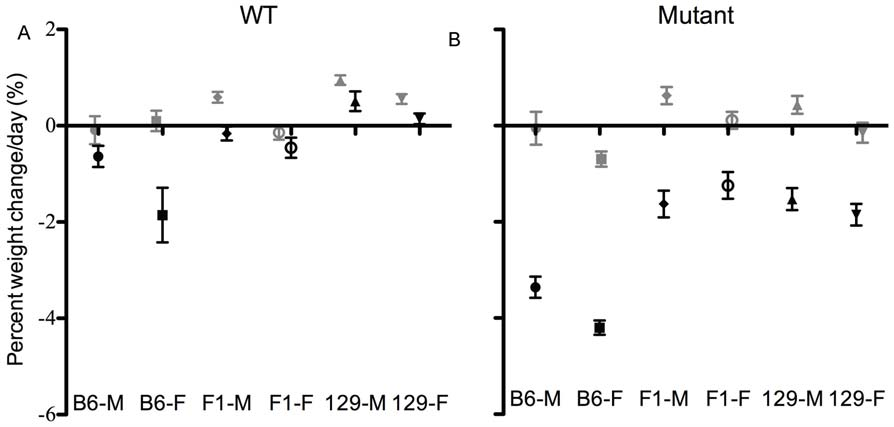
ATP8B1 mutant B6 mice lose substantial weight upon cholate feeding. Weight change per day in WT (A) and ATP8B1 mutant (B) mice of B6, 129, and F_1_ backgrounds after feeding of control (grey) or cholate (black) diet for 4–8 days; means and SEM are shown. N's for cholate diet: B6 (n = 8−13), 129 (n = 18−35), and F_1_ (n = 13−18); and control diet: B6 (n = 5−9), 129 (n = 22−29), and F_1_ (n = 11−16).

Rate of weight change per day was affected by genotype (ATP8B1 mutant mice lost more weight than WT), strain (B6 mice lost more weight than 129 and F_1_ mice), diet (mice lost more weight on cholate diet), and sex (females lost more weight, or gained less, than males); there are also several interactions (Table 1; Figure 2). On cholate diet, mutant mice of all strains lose weight (p<0.001 for all comparisons of mice on control versus cholate diet, except p<0.01 for F_1_ females; Figure 2b). The weight loss is greatest in mutant B6 mice, as compared to mutant 129 and F_1_ mice (p<0.05 to <0.001).

Among WT mice, only B6 females lost weight on cholate, as compared to control, diet (p<0.001; Figure 2a). This finding indicates that cholate diet may have a greater negative impact in B6 than in the other strains, even in the absence of the *Atp8b1* mutation.

### *Atp8b1* Mutation Results in Lowered Serum Cholesterol in B6 Mice

**Figure 3:**
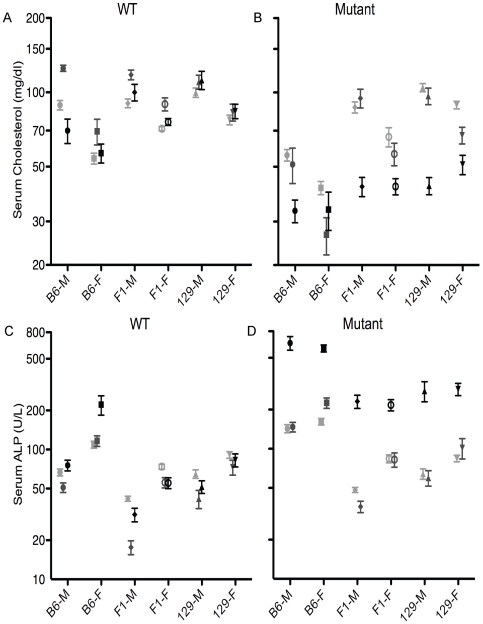
*Atp8b1* mutation results in lowered serum cholesterol and increased serum alkaline phosphatase (sALP) levels in B6 mice. Serum cholesterol and alkaline phosphatase levels in WT (A, C) and mutant (B, D) mice of B6, 129, and F_1_ backgrounds at baseline (light grey) and after feeding of cholate (black) or control (medium grey) diet for 4–8 days; means and SEM are shown. N's at baseline: B6 (n = 7−15), 129 (n = 42−66), and F_1_ (n = 21−36); N's for cholate diet: B6 (n = 5−12), 129 (n = 15−29), and F_1_ (n = 6−19) and for control diet: B6 (n = 5−12), 129 (n = 18−29), and F_1_ (n = 11−18).

At baseline, factor analysis showed overall effects of genotype (mutants<WT), strain (B6<129 and F_1_), and sex (females<males) on serum cholesterol levels (Table 1). In addition, there is a strain-genotype interaction; in B6 mice only, serum cholesterol was lower in mutants than in WT (males: p<0.001; females p<0.05) (Figure 3 a and b). Among sex-matched WT mice, serum cholesterol was similar between strains, except for B6 females, which had lower levels than 129 and F_1_ females (p<0.001; Figure 3a). Among sex-matched mutants, B6 mice had lower cholesterol levels than 129 mice (p<0.001, males and females); F_1_ mice were intermediate (Figure 3b).

### After Challenge with Cholate-Supplemented Diet, ATP8B1 Mutant Mice of All Strains Have Low Serum Cholesterol

Genotype, strain, and sex have similar effects on post-diet serum cholesterol levels, as at baseline. Overall, cholate feeding lowers serum cholesterol levels in ATP8B1 mutant mice compared to control-fed mutants, and there are diet-genotype and diet-sex interactions (Table 1). Cholesterol levels were significantly reduced in male mutants of 129 and F_1_ backgrounds after consumption of cholate, as compared to control, diet (p<0.001 for both), but not in the groups that had low cholesterol levels after consumption of control diet as well: B6 males, or females of all backgrounds (Figure 3b).

In contrast, among WT mice, only B6 males had lower cholesterol after consumption of cholate, than control, diet (p<0.001). In WT mice, no strain differences were apparent fter control diet, but after cholate diet, B6 mice had lower cholesterol than 129 mice (males: p<0.05; females: p<0.01) (Figure 3a).

### *Atp8b1* Mutation Results in Increased Serum Alkaline Phosphatase (sALP) Levels in B6 Mice

At baseline, genotype (mutant>WT), strain (B6>129 and F1), and sex (females>males) affect sALP levels (Table 1; Figure 3c and d). Serum ALP levels are higher in mutant B6 mice, as compared to WT (p<0.001, males and females), but there is no difference between mutant and WT mice of the other two strains (genotype-strain interaction). Mutant B6 mice had notably higher sALP levels than did sex-matched mutant mice of other backgrounds (p<0.001, each comparison).

### After Challenge with Cholate-Supplemented Diet, sALP Increases in ATP8B1 Mutant Mice of All Strains

After dietary challenge, there are overall effects of genotype (mutant>WT), strain (B6>129>F1), diet (cholate>control) and sex (females>males). Cholate feeding increases sALP levels in mutant mice (diet-genotype interaction; p<0.05 to <0.001) (Table 1; Figure 3d). Cholate-fed mutant mice of all strains had higher sALP levels as compared to cholate-fed WT mice (p<0.001 for all comparisons; Figure 3c and d). In mutant mice after either diet, B6 mice have notably higher sALP than do 129 or F_1_ mice (p<0.001 for all comparisons except <0.01 for male B6 versus 129). In WT mice after cholate diet, B6 females have higher levels than do females of 129 or F1 background (p<0.001). There are also diet-sex and sex-genotype interactions.

### Serum Bilirubin Concentration Increases in ATP8B1 Mutant Mice after Consumption of Cholate-Supplemented Diet

**Figure 4:**
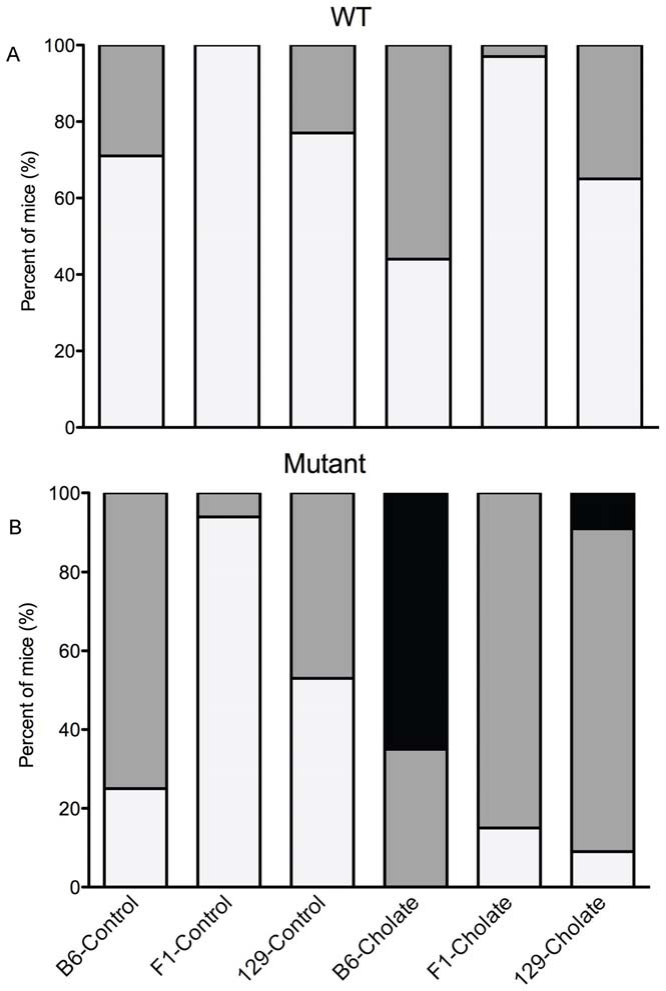
Serum bilirubin concentration increases in ATP8B1 mutant mice upon cholate feeding. Proportion of WT (A) and ATP8B1 mutant (B) mice of B6, 129, and F_1_ backgrounds with normal (light grey), moderately elevated (medium grey), and highly elevated (black) serum bilirubin levels in after feeding cholate or control diet for 4–8 days. N's for cholate diet: B6 (n = 5−8), 129 (n = 30−31), and F_1_ (n = 13−25); and control diet: B6 (n = 10−13), 129 (n = 39−48), and F_1_ (n = 18−23).

At baseline, no differences in bilirubin levels between groups of mice were detected (Table 1; values not shown). Factor analysis of post-dietary challenge data showed an overall effect of genotype, strain and diet, and a diet-genotype interaction. For all strains, mutant mice have higher serum bilirubin levels than WT mice when fed cholate diet (p<0.001); after consumption of control diet, the effect of genotype is significant only for the 129 strain (p<0.05; Figure 4). After cholate diet, serum bilirubin was higher in B6, than 129 or F_1_, mutant mice (p<0.001; Figure 4b). In contrast, for mutant mice after control diet, and WT mice after both diets, serum bilirubin levels in B6 and 129 strains were similar, and higher than those seen in F_1_ mice (p<0.001, each comparison). Mutant, but not WT, mice of all strains have higher bilirubin levels after cholate diet as compared to control diet (p<0.001; diet-genotype interaction).

### Serum Bile Salt Levels Are Higher in ATP8B1 Mutant Mice

**Figure 5:**
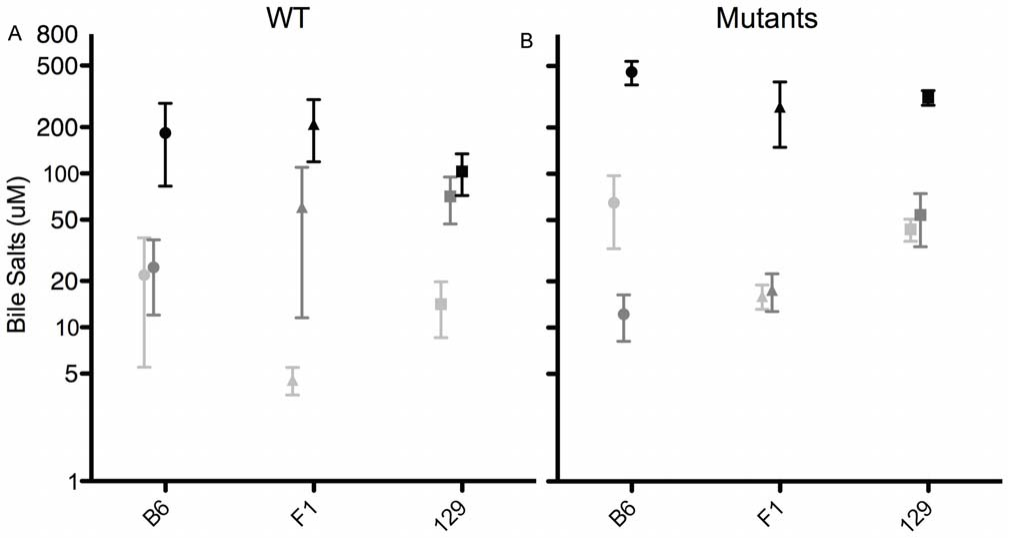
Serum Bile salts levels are higher in ATP8B1 mutant mice. Serum bile salt levels in WT (A) and Atp8b1 mutant (B) mice of B6, 129, and F_1_ backgrounds at baseline (light grey) and after feeding of cholate (black) or control (medium grey) diet for 4–8 days; means and SEM are shown. N's at baseline: B6 (n = 9−15), 129 (n = 48−43), and F_1_ (n = 43−45). N's for cholate diet: B6 (n = 7−13), 129 (n = 18−35), and F_1_ (n = 13−18); and control diet: B6 (n = 5−9), 129 (n = 22−29), and F_1_ (n = 11−16).

At baseline, factor analysis showed overall effects of mutation (mutants>WT; 129: p<0.001; F_1_: p<0.01; B6: p<0.05) and strain (B6 and 129>F_1_) (Table 1, Figure 5). B6 mutant mice had higher serum bile salt levels than did F_1_ mutant mice (p<0.05; Figure 5b). After dietary challenge, there is an overall effect of genotype and a genotype-diet interaction (Table 1, Figure 5). Mutant mice fed cholate diet have higher serum bile salt levels as compared to those fed control diet (p<0.001, each comparison); in WT mice, effect of diet is smaller (B6 and F_1_: p<0.05; 129: ns). Factor analysis did not identify an overall effect of strain after dietary challenge. Specifically after cholate feeding, however, serum bile salts were modestly higher in mutant mice of B6 and 129 strain background, than in those of F_1_ background (p<0.05, both comparisons).

### Gallbladder Bile Composition after Consumption of Cholate Diet Is Influenced by *Atp8b1* Mutation and Strain

Bile was more amenable to collection by needle aspiration from mice after cholate diet, as they tended to have well-filled gallbladders, than from mice after control diet, whose gallbladders often contained very little fluid. Therefore, we analyzed bile composition after cholate feeding only.

**Figure 6:**
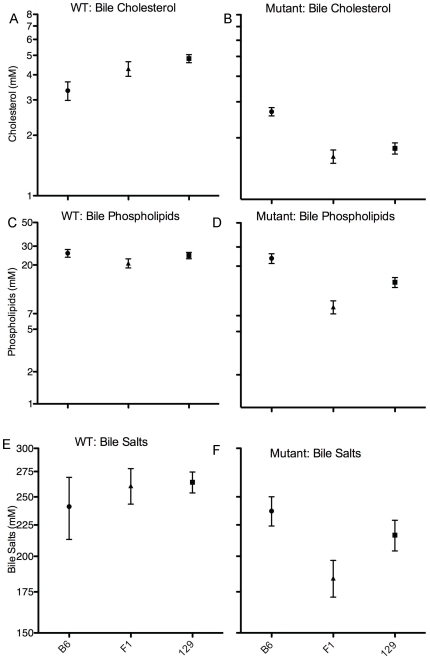
Composition of gallbladder bile after feeding of cholate-supplemented diet is influenced by ATP8B1 mutation and strain. Bile cholesterol, phospholipid, and bile salt levels in WT (A, C, & E) and ATP8B1 mutant (B, D, & F) mice of B6, 129, and F_1_ backgrounds after feeding of cholate diet for 4–8 days; means and SEM are shown. N's: B6 (n = 11−19), 129 (n = 39−52), and F_1_ (n = 19−22).

For concentrations of cholesterol and phospholipids in bile, there were overall effects of genotype (mutant<WT) and strain (B6>129 and F_1_), and for cholesterol, a strain-genotype interaction (Table 1; Figure 6 a–d). 129 and F_1_, but not B6, mutant mice had lower biliary cholesterol and phospholipids concentrations than strain-matched WT mice (p<0.01 to <0.001; Figure 6a–d). Mutant mice of B6 background had higher cholesterol and phospholipid concentrations than did mutant mice of F_1_ or 129 backgrounds (p<0.01 to <0.001; Figure 6b, d). In contrast, for WT mice, B6 mice had lower cholesterol than did 129 mice (p<0.05: Figure 6a). Factor analysis identified an effect of genotype (mutants<WT) on biliary bile salt concentration, which attained significance in the 129 strain (p<0.05; Figure 6e, f).

### ATP8B1 Mutant B6 Mice Have Larger Livers than Mutant 129 Mice

**Figure 7:**
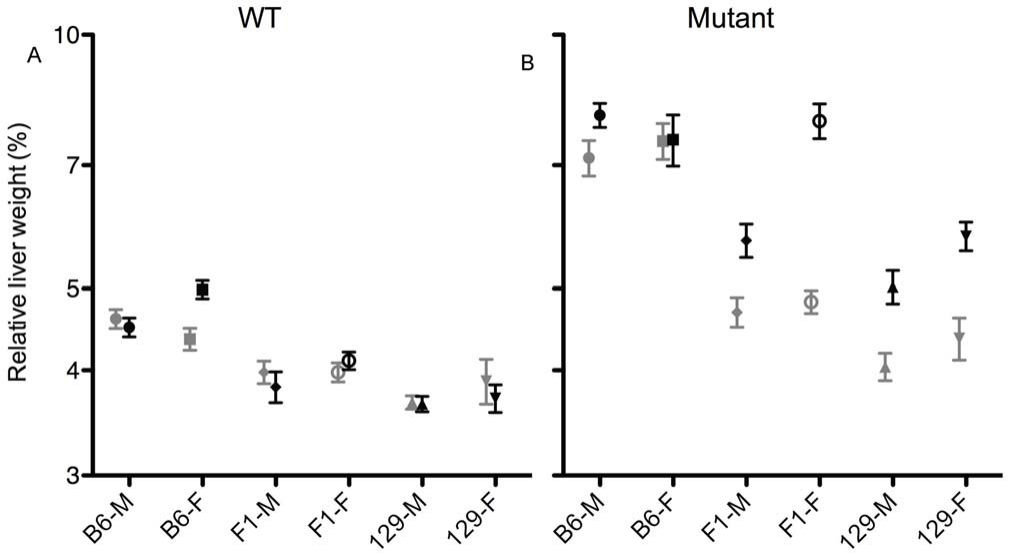
ATP8B1 mutant B6 mice have larger livers than mutant 129 mice. Liver weight as a proportion of final body weight in WT (A) and ATP8B1 mutant (B) mice of B6, 129, and F1 backgrounds after feeding of cholate (black) or control (grey) diet for 4–8 days; means and SEM are shown. N's for cholate diet: B6 (n = 7−13), 129 (n = 18−35), and F1 (n = 13−18); and control diet: B6 (n = 5−9), 129 (n = 22−29), and F1 (n = 11−16).

There were overall effects of genotype (mutant>WT), strain (B6>129, with F_1_ intermediate), diet (cholate>control), and sex (females>males) on liver weight as a proportion of final body weight. We also detected genotype-strain, genotype-diet, and strain-diet interactions, and a 3-way interaction between strain, diet, and genotype (Table 1, Figure 7). Livers of mutant mice were larger than those of sex-matched WT mice for B6 and F_1_ mice fed control (B6: p<0.001; F_1_: p<0.05) and cholate diet (p<0.001 for all), but in 129 mice this difference was only seen after cholate diet (p<0.001). After consumption of control diet, mutant mice of B6 background had larger livers than those of 129 or F1 backgrounds (p<0.001, all comparisons; Figure 7b). After cholate diet, livers of mutant 129 and F_1_, but not B6, mice are enlarged relative to after control diet (p<0.001, except F_1_ males: p<0.05). Nevertheless, liver weights in male mutant mice after cholate diet consumption were still higher in B6, than in 129 or F_1_, mice (p<0.001, both comparisons); in female mutants after cholate feeding, B6 and F_1_ mice have larger livers than 129 mice (B6 versus F_1_: p<0.01; 129 versus F_1_: p<0.001; Figure 7b). In WT mice after either diet, livers of B6 mice trended slightly larger than in 129 and F_1_ mice (control diet: p<0.001 for B6 versus 129 males, ns for others; cholate diet: p<0.05 to p<0.001; Figure 7a).

### Hepatic Levels of Triglycerides and Cholesterol Are Influenced by Diet and *Atp8b1* Mutation

Triglycerides (TG), total cholesterol (TC), and free cholesterol (FC) were quantified in snap-frozen liver tissue from a representative subset (143 mice) of the study sample; amount of esterified cholesterol (EC) was calculated by subtracting FC from TC.

**Figure 8:**
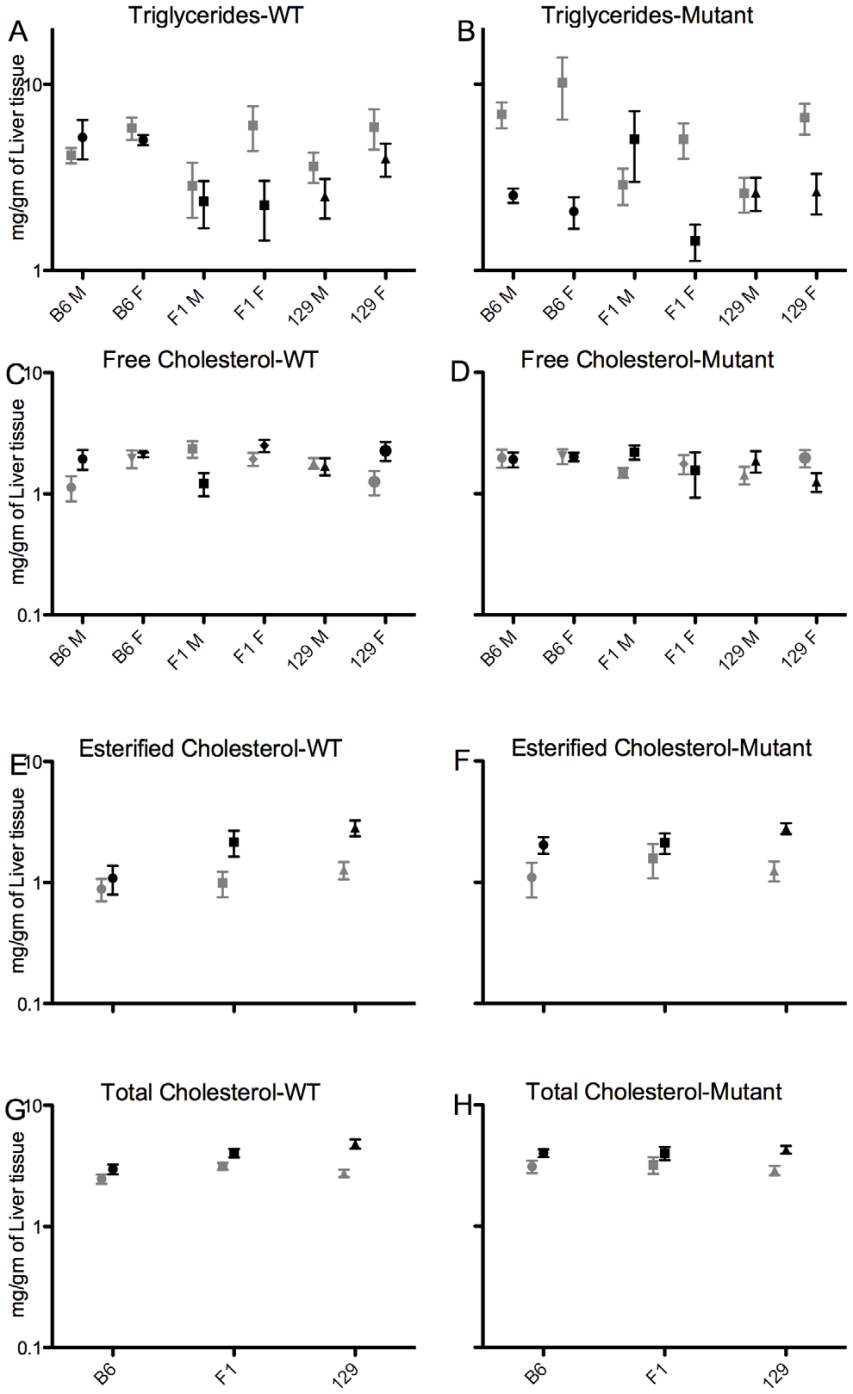
Hepatic levels of triglycerides and cholesterol are influenced by diet and *Atp8b1* mutation. Hepatic triglycerides and cholesterol in WT (A, C, E, and G) and mutant (B, D, F, and H) mice of B6, 129, and F1 background after cholate (black) and control (grey) diet for 4–8 days; mean and SEM are shown. N's for cholate diet: B6 (n = 4−10), 129 (n = 8−20), and F1 (n = 4−9); and control diet: B6 (n = 4−11), 129 (8−19), F1 (n = 4−8).

No overall effects of genotype, diet, strain, or sex were detected for TG and FC; however, for both, diet-genotype and diet-genotype-sex interactions were detected (Figure 8A–D). TG were lower in B6 mutant females after cholate diet than in both B6 mutant females after control diet (p<0.01) and B6 WT females after cholate diet (p<0.05).

An overall effect of diet (cholate>control) was detected for TC and EC. After consumption of cholate diet, TC was higher in 129 WT mice (p<0.05), and EC was higher in 129 mutant mice (p<0.01), than respective groups after control diet (Figure 8E–H). Although factorial analysis did not detect an overall strain effect, after cholate diet, 129 WT mice had higher EC than B6 WT mice (p<0.05).

## Discussion

We have previously shown that mice lacking Atp8b1 exhibit a mild form of human ATP8B1 deficiency, but do not suffer from progressive cholestatic liver disease [12]–[14], [16]; most of this work was performed in 129 mice. Atp8b1 mice were previously found to exhibit some of the characteristic phenotypic features of human ATP8B1 deficiency, such as elevation of bile salts, bilirubin and liver enzyme activities in serum, when fed a diet supplemented with 0.5% cholate. Results from the analyses presented here indicate strain-genotype interaction; the manifestation of many phenotypic features in ATP8B1 mutant mice depends on strain background.

ATP8B1 mutant B6 mice manifest a number of phenotypes that have correlates in human ATP8B1 deficiency, and are not detected, or less readily apparent, in the 129 strain background. In the B6 strain, *Atp8b1* mutation is associated with lower serum cholesterol, higher sALP, and higher serum bilirubin, mirroring findings in human ATP8B1-deficient patients [18]–[20]. Similarly, slow growth during the nursing period, and substantial weight loss on cholate diet, phenotypes reminiscent of the failure-to-thrive seen in human patients, are most notable in mutant mice of B6 background. Mutant mice of B6 background also exhibit greater hepatomegaly. Results of the hepatic lipid assays performed, as well as histological evaluation of a subset of study samples (data not shown), indicate that this enlargement is not due to lipid accumulation, in agreement with findings in human ATP8B1-deficient patients. Instead, histologic assessment of mitotic activity (data not shown) suggests that B6 mutant mice may have increased cell proliferation, relative to 129 and F1 mutant mice. With respect to these phenotypes, the ATP8B1 mutant mouse in the B6 background strain may be a better model of human ATP8B1 deficiency than is the mutant mouse in the 129 strain.

A consistent finding in our study was that serum cholesterol was decreased in mutant, as compared to WT, mice in the B6 background; after cholate feeding, this mutation effect was present in all strains. It has been well-established both in mice and humans that cholestasis leads to a decrease in HDL and its main apolipoprotein apoA1 [21], [22]. On the other hand, some forms of chronic cholestasis are associated with increased cholesterol in the VLDL/LDL fraction, at least partly due to the appearance of Lipoprotein X (LpX) in the serum [23]. Formation of LpX critically depends on the canalicular transporters responsible for biliary lipid secretion and LpX is not found in states of cholestasis caused by transport defects, including PFIC [24], [25]. In addition, in mice the majority of serum cholesterol is in HDL, and therefore decreased HDL formation in cholestasis will have a lowering effect on total serum cholesterol. Feeding of a cholate-supplemented diet aggravates the intrahepatic cholestasis, which will lead to a further reduction of serum cholesterol.

Results for most of the evaluated phenotypes indicate greater abnormalities in mutant mice of B6, as compared to 129, strain background; however, regarding bile composition, we detect differences between WT and mutant mice of 129 background that are not apparent in the B6 strain. We have previously reported that ATP8B1 mutant mice characteristically have increased biliary secretion of cholesterol compared with WT of the same genetic background [13], [14]. This enhanced secretion occurs independent of the function of Abcg5/8 and therefore most likely represents direct extraction of cholesterol due to a reduced resistance of the canalicular membrane to the detergent action of bile salts. In the present study, we find reduced concentration of cholesterol (and phospholipids) in bile of mutant mice of 129 and F_1_ background. These contrasting results are likely due to important differences between these studies. In previous studies, we evaluated hepatic bile secretion after acute infusion of taurocholate, while the current study is focused on composition of gallbladder bile after chronic challenge by feeding of a cholate-supplemented diet. Based on these observations, we hypothesize that the increased cholesterol secretion occurs only during acute bile salt challenge, in mutant mice of 129 and F_1_ background. Cholesterol extraction from the canalicular membrane subsequently leads to decreased membrane cholesterol content and cholestasis, also reducing the normal Abcg5/8 mediated cholesterol secretion into bile [26]. Hence, in a chronic situation reduced cholesterol secretion may be observed, as opposed to increased cholesterol secretion in an acute situation of bile salt infusion.

Our findings imply the existence of modifier loci regulating the ATP8B1 mutant phenotype; the presence of similar loci in people may underlie, at least in part, the varying severity and nature of disease manifestations that can be seen, even between patients carrying the same, or similar, ATP8B1 mutations [9]. For many of these phenotypes, mutant F_1_ and 129 mice are similar to each other, while mutant B6 mice differ, and are more abnormal. These findings suggest that susceptibility to cholestasis-related phenotypes in these mice is recessive; however such determination can only be made after studying the phenotypes in an experimental cross. Genetic mapping studies of Atp8b1 mutant mice, employing an intercross, or a backcross onto B6 background, will allow us to identify modifier loci of ATP8B1 mutant phenotypes. Mapping of such loci in mice, and correlating of results with human genotype-phenotype studies, may provide novel insight into the function of ATP8B1 and the biological mechanisms of ATP8B1 deficiency in humans.

We have also identified strain-dependent differences in WT mice apparent at baseline and/or after dietary challenge. B6 WT mice have lower serum cholesterol (both sexes), and higher sALP (females), as well as greater weight loss (both sexes), and more enlarged livers (both sexes) than do WT 129, and sometimes F_1_, mice. Some of these findings are magnified upon cholate feeding, suggesting a greater innate sensitivity to cholate feeding in the B6, than 129, strain. Our findings may have general implications for choice of strain when studying hepatobiliary phenotypes, either in WT mice, or in mice with targeted mutations in other genes influencing hepatobiliary phenotypes.

## Materials and Methods

All mice were maintained in a specific-pathogen-free animal facility in San Francisco; studies were conducted under a protocol approved by the UCSF IACUC. The embryonic stem cell line used in generation of the mice was derived from the 129S4 strain, and mice of the genetically most closely related commercially available 129 substrain, 129S1, were subsequently used for breeding [12]. We therefore backcrossed the Atp8b1 mutation separately onto the B6, 129S1, and 129S4 strains. The studies reported here were performed during the course of this backcrossing. For B6, the first experiments were performed on mice after 5 generations of backcrossing, with a median of 12 backcross generations for phenotyped mice. For phenotypes presented here, little or no effect of 129 substrain (129S1 versus 129S4) was detected, so data for these 129 substrains were pooled. A minority of WT mice was of pure strain stock, rather than derived from backcrossing. For F_1_ mice, similar numbers were generated with B6 as the paternal, and as the maternal, strain.

At approximately 21 days after birth, pups were weaned, weighed and tagged. For many litters born to heterozygote couples, pups had also been weighed at approximately day 10, and uniquely identified at that time, so that weight gain during the 2^nd^ half of the nursing period could be evaluated; we did not want to disturb the litters earlier than that, to avoid increasing the risk of mothers abandoning their litters. To account for effects of factors such as litter size and precise age in days on pup weight, weights of mutant pups were normalized to those of WT and heterozygous littermates.

Mice were aged a minimum of 3 months prior to study diet administration (Dyets, Inc. catalog number 101914±0.5% cholic acid, sodium salt [Calbiochem]; a small, initial pilot study was performed with a highly similar diet [K4068.02, Arie Blok Diervoeders, Woerden, The Netherlands]). Mice were anesthetized, and a ‘baseline’ blood collection was performed. Then, standard mouse chow was replaced with control or cholate-supplemented diet. To optimize the number of days mice would be on study diet, we assessed impact of diet administration for varying lengths of time, monitoring mouse body weight and condition. Mutant B6 mice could not consistently remain on the cholate diet for >6 days without demonstrating excessive weight loss. Therefore, >95% of the mice in this study underwent dietary challenge for 6–7 days. To make fullest use of data from animals studied while optimizing diet length, we assessed impact of number of days on diet on phenotypes. Regression analysis did not identify differences attributable to number of days on diet over 4–8 days, so data from all mice on the diet for 4–8 days were pooled for analysis. In total, results from 396 mice are included in this study, tallied by strain as follows: 72 B6, 108 129S1, 100 129S4, and 116 F_1_ (the latter including mice derived from crossing B6 with 129S1, and B6 with 129S4).

We established a standard protocol in which mice were fasted for ≥4 hours, then anesthetized, and blood, gallbladder bile, liver, and spleen (for isolation of DNA to confirm genotype) were collected at sacrifice. Serum ALP, cholesterol, and bilirubin were assayed in a clinical laboratory. Serum bile salts were assayed using the Total Bile Acid Assay kit (DZ042A-K, Diazyme Labs, USA). Cholesterol, phospholipids, and bile salts in gallbladder bile were assayed as previously described [27]. Free cholesterol, total cholesterol, and total triglycerides were measured in lipid extracts from snap-frozen liver tissue using commercial assay kits (Wako Free Cholesterol E [435-35801, Wako Diagnostics]; Infinity cholesterol reagent [TR13521, Fisher Diagnostics]; Infinity triglyceride reagent [TR22321, Fisher Diagnostics]).

### Statistical Analysis

Since 24 factorial combinations are present, there were 276 possible ways of grouping the mice into two groups based on their mutation status, genetic background, diet and sex. To reduce the number of comparisons examined, and simplify the process of determining which factors affect a trait of interest, we adopted the following procedure: For each trait of interest, we fit a full factorial model with all main effects, two-factor, three-factor and the four-factor interaction [17]. Then we performed backward selection using the Bayesian Information Criterion [28]. This approach balanced the explanatory power of the model against model complexity, yielding a parsimonious list of factors that influenced the trait of interest. Lower order terms were always included if a higher-order interaction was present. For most phenotypes we used linear regression to fit the models. Serum bilirubin was analyzed as an ordinal variable. Baseline bilirubin data were binned into 2 levels, normal (<0.1 mg/dl) and elevated (≥0.1 mg/dl), and analyzed using a binomial linear model. Post-diet data were binned into 3 levels, normal (<0.1 mg/dl), moderately elevated (0.1–2 mg/dl), or highly elevated (>2 mg/dl), and analyzed using proportional odds models. To reduce co-linearity in the proportional odds model, we used ridge regression, using the equivalent of one mouse with no association with any of the factors. These analyses were designed using the R programming language [29]; for the annotated code, see Supplementary Files S1, S2, S3.

P-values reported in the text are derived from analysis of variance (ANOVA) with Tukey's post-test (performed on the relevant sub-groups), or the chi-square test. These analyses were performed using PRISM 5.0 (Graphpad Software, Inc.) or programmed in Excel (Microsoft Corp.). Except for serum bilirubin and rate of weight loss, data were log-transformed for all analyses. (As serum bile salt levels were undetectable in a small number of mice, and therefore recorded as zero, we added 0.1 prior to log transformation.) For subtle effects, significance is occasionally obtained in the factorial analysis, but not in ANOVA, due to differences in power.

## Supporting Information

File S1 (0.00 MB TXT)

File S2 (0.01 MB TXT)

File S3 (0.01 MB TXT)
